# Trace Level Detection of Bisphenol A Analogues and Parabens by LC-MS/MS in Human Plasma from Malaysians

**DOI:** 10.1155/2020/2581287

**Published:** 2020-04-29

**Authors:** Idha Arfianti Wiraagni, Mustafa Ali Mohd, Rusdi Abdul Rashid, Didi Erwandi bin Mohamad Haron

**Affiliations:** ^1^Department of Pathology, Faculty of Medicine, University of Malaya, Kuala Lumpur, Malaysia; ^2^Department of Forensic Medicine and Medicolegal, Faculty of Medicine, Universitas Gadjah Mada, Yogyakarta, Indonesia; ^3^Shimadzu-UM Center for Xenobiotics Studies (SUCXeS), Faculty of Medicine, University of Malaya, Kuala Lumpur, Malaysia; ^4^Department of Psychological Medicine, Faculty of Medicine, University of Malaya, Kuala Lumpur, Malaysia

## Abstract

In this study, a novel LC-MS/MS method was designed using a simple extraction procedure that was scientifically developed to capture the most relevant bisphenol A (BPA) analogues (BPB, BPF, BPS, and BPAF) and parabens (propylparaben, ethylparaben, butylparaben, and methylparaben) in human plasma. The LC-MS/MS method was validated using US FDA guidelines, and all validation requirements were satisfactory. This is the method that allows for the detection of plasma bisphenols and parabens in one run and is also the fastest BPA analogue and paraben detection technique for human plasma. The method was used to analyze samples from 150 healthy volunteers from Malaysia who enrolled in the study. No BPB was detected in any of the volunteers; however, 99.3% were positive for BPF. Only 24% and 10.7% of volunteers were positive for BPAF and BPS, respectively. A high percentage of volunteers were negative for propylparaben, ethylparaben, butylparaben, and methylparaben (56%, 68%, 86.7%, and 83.3%, respectively). These results suggest that persons in Malaysia are exposed to different BPA analogues and parabens, from both the daily use of products (cosmetic and plastic products) and the environment.

## 1. Introduction

Bisphenol is an industrial chemical that has been used in the manufacturing of plastics and resins since the 1960s. It is used in the industry to increase the thickness and durability of materials. Bisphenol is commonly found in polycarbonate plastic, food storage containers, reusable drink containers, children's toys, and canned foods. It is released from consumer goods, leading to its detection in waste water, drinking water, soil, dust, food, and air [1]. Bisphenol B (BPB), BPS, BPF, and BPAF were analyzed in this study. Bisphenols are mostly soluble in ethanol, acetone, methanol, benzene, chloroform, and ether [2].

Bisphenols are absorbed rapidly via the gastrointestinal tract. They are metabolized in the liver and rapidly conjugated with uridine diphosphate-glucuronic acid (UDP-glucuronic acid), forming bisphenol glucuronide. Bisphenol glucuronide is rapidly formed and excreted via urine, feces, or bile, with a half-life about 5.3 hours (h). All of these processes are completed within 24 h [3]. In pregnant women, the fetus and newborn can also be exposed to bisphenol from the mother via placental transfer and milk [4].

Parabens have simple esters that possess very effective antimicrobial and antifungal properties. Methylparaben, ethylparaben, propylparaben, and butylparaben were analyzed in this study [5]. Products found to contain parabens include hand soap, body lotion, shampoo, conditioner, cosmetics, hair spray, toothpaste, jams, jellies, fillings, and toppings [6]. In the environment, parabens have been detected in urban streams, rivers, and drinking water sources [7]. Parabens are odorless and tasteless (or they numb the tongue) and are mostly soluble in ethanol, water, methanol, acetone, and ether [2].

The main route of exposure to parabens is through oral ingestion and via skin penetration. Parabens are quickly metabolized by the liver (through esterase hydrolysis and glucuronidation by several UDP-glucuronosyltransferase isoforms), the plasma, and the skin. Hydrolysis in the microsomes of the human liver is conducted more rapidly than that in the plasma. They are then converted into p-hydroxybenzoic acid and excreted in urine. The extensive use of personal care products contributes significantly more to the high paraben concentrations compared to oral ingestion. Metabolism by the intestine and liver is fast following oral exposure to parabens [8].

BPA analogues and parabens are similar to endogenous estrogen, which have the ability to bind to estrogen receptors, stimulate estrogen production, and also alter gonadotrophin hormone secretion [9]. These mechanisms can stimulate the development of endometriosis [9]. They can also inhibit the estrogen-inactivating enzyme 17*β*-hydroxysteroid dehydrogenase [8]. At the cellular level, there are some studies that report that parabens can disrupt cellular function and cause chromosomal aberration [6]. BPA analogues and parabens are lipophilic pollutants that are known to bioaccumulate in fatty tissue. They can accumulate in human breast tumors at a mean concentration of 20.6 ± 4.2 ng/g tissue [6]. In addition, a study of more than 1,800 persons from the US reported an inverse association between paraben concentration and circulating thyroid hormone levels in adults, mainly in females [10].

For the biologic monitoring of BPA analogues and parabens in humans, some studies used various matrices such as placental tissue, tumor tissue, urine, serum, semen, plasma, amniotic fluid, breast milk, follicular fluid, saliva, and umbilical cord blood [11]. Our research question was how to perform LC-MS/MS analysis of bisphenols and parabens using a simple extraction procedure in one run. Analytical techniques used for measuring bisphenol and parabens in human matrices are GC-MS, LC-MS, LC-MS/MS, and enzyme-linked immunosorbent assay [12].

## 2. Methods

This research study analyzes the presence of BPA analogues and parabens in 150 healthy Malaysian volunteers who provided consent for the study. The volunteers were recruited from University Malaya from July 2019 to October 2019. Demographic details such as gender, age, ethnicity, education, occupation, health status, social habits, eating habits, and daily habits such as cosmetic use were recorded. We tested for the following in human plasma: BPB, BPS, BPF, BPAF, methylparaben, ethylparaben, propylparaben, and butylparaben using a validated method [13]. The inclusion criteria were 18 years of age or older, being fully conscious, being physically healthy, no current serious psychiatric symptoms (i.e., psychotic episode), and no current legal problems. The exclusion criteria were refusal to participate in the research, the need for advanced medical attention for a serious illness, the need for psychiatric care to treat psychiatric symptoms, and the patient being an immediate danger to themselves/others. Institutional approval for the analysis of human samples was obtained from the Ethical Committee of the University Malaya Medical Centre.

The bisphenol and paraben standards were purchased from Sigma-Aldrich (St. Louis, MO, USA). All solvents and reagents used were of HPLC grade and purchased from Merck (Darmstadt, Germany). In this research, the LC system consisted of an LC-20AD XR UFLC system with a SIL-HT automatic sample injector (Shimadzu, Kyoto, Japan). The analytical column used was a Phenomenex Gemini-NX C18 (150 mm length × 2.0 mm ID, particle size 5 *μ*m) and Phenomenex Gemini-NX C18 guard column (4 mm ID × 2.0 mm length). The column temperature was 40°C with a total running time of 11 min. Mobile phases used were 2 mM ammonium acetate in water (pH 6.7) in pump A and methanol in pump B. The flow rate was set at 0.35 mL/min, and a gradient elution was used at room temperature. The gradient program began with 20% B, then ramped to 98% B at 6.00 min, and was held for 9.00 min. The gradient then returned to 20% B at 9.01 min, and this condition was held for a further 11.00 min. Sample injection volume was 2 *μ*L. A Linear Ion Trap Quadrupole LC-MS/MS Spectrometer, QTRAP 5500, fitted with an ESI probe was used to perform mass spectral analysis. The LC-MS/MS system was controlled by the software called Analyst, version 1.6.3 (Applied Biosystems). Nitrogen was used as the nebulizer, auxiliary, collision, and curtain gas. This machine was operated in the negative ionization mode for bisphenols and parabens.

### 2.1. Validation Study

**Figure 1:**
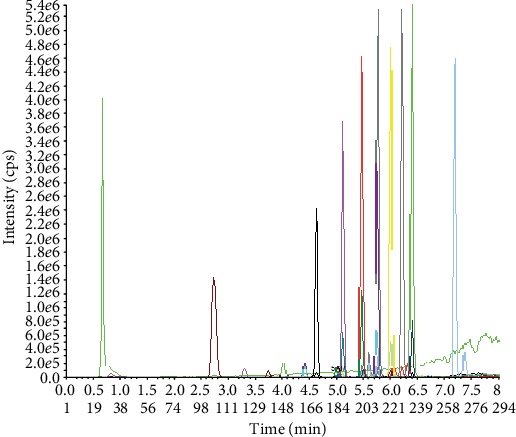
BPA analogue and paraben (spiked) plasma, at a concentration of 50 ng/mL.

This method was validated for selectivity, specificity, linearity, accuracy, precision, recovery, and stability according to the principles of bioanalytical method validation guidance for industry (US FDA guidelines). This method has good specificity and selectivity because the blanks showed no area values higher than 15% of the analyte retention time. There was no crossinterference of the retention time where the external standard appears (Figure 1).

Seven calibration points were used to evaluate the linearity of the standard calibration curve. The calibration curve showed good linearity with a regression coefficient (*R*) value of interday analyses over seven calibration points of greater than 0.996. The standard curve was linear with a 1/*x* weighing factor [14]. For bisphenols, the lower limit of detection (LOD) was established at 5 ng/mL and the lower limit of quantitation (LOQ) was established at 10 ng/mL, with an average signal to noise ratio > 10. For parabens, the lower limit of detection (LOD) was established at 0.5 ng/mL, while the lower limit of quantitation (LOQ) was established at 1 ng/mL, with an average signal to noise ratio > 10 [15].

Based on the mean coefficient of variation (%CV) for three quality control (QC) samples, we produced precision. These QC samples were prepared by spiking aliquots of drug-free plasma with a mixed bisphenol working solution standard and a mixed paraben working solution standard to produce a concentration of 1 *μ*g/mL of parabens and 10 *μ*g/mL of bisphenols (in one mixture). All QC samples were prepared by diluting a mixed endocrine disrupting chemical (EDC) (bisphenols and parabens) stock plasma standard into different volumes of plasma to obtain three QC samples. For bisphenols, three QC samples at the concentrations of 70, 800, and 1,500 ng/mL were prepared. For parabens, three QC samples of 7, 80, and 150 ng/mL were prepared. The intraday precision of bisphenols in the plasma samples ranged from 2.07% to 9.87%. When analyzed using the three quality control samples, the accuracy of intraday bisphenols in plasma ranged from 87.72 to 111.7%. However, for parabens, the coefficient of variation and accuracy ranged from 1.45 to 9.78% and 87.86 to 112.57%, respectively. The results from this study clearly indicate that the method that was developed for the measurement of bisphenols and parabens in plasma is accurate, precise, and reproducible for the quantification within the same day.

The interday precision of bisphenols in plasma samples ranged from 5.01% to 11.9%. However, when analyzed using the three quality control bisphenols in samples, the accuracy for interday plasma samples ranged from 91.77 to 106.8%. The coefficient of variation and accuracy of parabens ranged from 3.87 to 8.49% and 93.86 to 108.43%, respectively. The results showed that the method that was developed to measure bisphenols and parabens in plasma is accurate, precise, and reproducible.

Recovery was calculated as a percentage of the measured area of the extracted spiked plasma sample over that of the peak area of the nonextracted standard. The recovery of bisphenols and parabens was tested at 10, 50, and 100 ng/mL, and it ranged from 84.6 to 102.7%, 91.67 to 103.57%, and 97.3 to 108.82%, respectively. The recovery of parabens ranged from 107.69 to 113.04%, 92.86 to 110.42%, and 92.31 to 99% at 10, 50, and 100 ng/mL, respectively. The results indicate that the extraction efficiency of bisphenols and parabens (using the protein precipitation method) was effective. To minimize the matrix effect, we used syringe filters and guard columns for the sample cleanup process.

This study had stability tests including freeze-thaw stability, bench top 4 h stability, 24 h autosampler stability, and long-term stability. The results of the stability tests showed that the bisphenols and parabens in plasma samples were stable during sample storage, extraction, and the chromatography processes.

## 3. Results and Discussion

This method was applied to the screening of BPA analogue and paraben levels in human blood plasma from 150 healthy volunteers from Malaysia. The majority of the participants were from West Malaysia (91.3%), specifically Kuala Lumpur and Selangor. The research subjects were 30.03 ± 8.64 years old on average, and the youngest was 20 and the oldest was 60 years old. The mean height and weight were 158.3 ± 8.48 cm and 63.78 ± 17.89 kg, respectively.

The result of BPA analogue and paraben analysis is presented in Table 1. For BPA analogues, no BPB was detected in any of the participants; however, most (99.3%) were positive for BPF. Only 24% and 10.7% of the volunteers were positive for BPAF and BPS, respectively. A high percentage of volunteers were negative for propylparaben, ethylparaben, butylparaben, and methylparaben (56%, 68%, 86.7%, and 83.3%, respectively).

From the mean number of volunteers positive for BPA analogues and parabens (Table 1), we can conclude that the concentration of BPF was higher compared to that of BPAF and BPS. For parabens, methylparaben had the highest concentration at 7.7 ± 3.91 ng/mL. The other parabens (propylparaben, ethylparaben, and butylparaben) were detected at less than 1.6 ng/mL. The main route of exposure to bisphenols and parabens is through oral ingestion and via skin penetration. Bisphenols and parabens are quickly metabolized by the liver, plasma, and skin. Hydrolysis in the microsomes of the human liver is conducted more rapidly than that in plasma. They are then converted to bisphenol glucuronide and *p*-hydroxybenzoic acid, followed by excretion via urine. If the urine contains higher levels of parabens and bisphenols, higher concentration will be observed in the plasma as well. The paraben levels and levels of BPAF and BPS might be so low in this study because after entering the body, the compound is rapidly metabolized by the liver and excreted in urine [8].

The results can be compared to the National Health and Nutrition Examination Survey (NHANES) conducted between 2013 and 2014 that analyzed urinary BPS and BPF levels in US adults and children and found that 89.4% were BPS positive and 66.5% were BPF positive among the subjects [16]. In another study that collected 315 urine samples from populations in the United States, Vietnam, Japan, China, India, Malaysia, Korea, and Kuwait, BPS was positive in 81% of the urine samples [17]. One study in human plasma detected BPF three times higher than BPA [18]. Research on the levels of BPA analogues is rarely conducted in samples other than urine. These results show that BPF, BPAF, and BPS are gradually replacing BPA.

However, with parabens, in one study in an urban community in western Canada, urinary levels of methyl-, ethyl-, propyl-, butyl-, and isobutyl parabens were detected, in which the highest concentration was that of methylparaben [5]. The same result was observed with urine samples obtained from 100 US adults. Methylparaben was detected in up to 96% of samples and had the highest median concentration (43.9 ng/mL) [19]. The highest concentration of methylparaben was also observed in one study in human plasma (27-50.000 ng/L) [20]. In a study with 60 healthy Danish men, methyl- and n-propylparabens were detected in the majority of serum and seminal plasma samples [21].

We performed statistical analyses to evaluate the significance of the differences in bisphenol levels, considering different social, demographic, daily, and eating habits. The independent sample *t*-test was conducted to compare the differences between two variables (Table 2). There were significant differences between the BPS levels that correlated with gender, BMI, and age (*p* = 0.003, *p* = 0.004, and *p* ≤ 0.001, respectively). Higher BPS levels were observed in males compared to females. The BPS levels were higher in participants ≥ 33 years old compared to those < 33 years old and higher in overweight individuals compared to those of normal weight. There were no significant differences among other bisphenols.

We performed statistical analysis to evaluate the differences in paraben levels based on different social demographics and cosmetic use. The independent sample *t-*test was used to compare the differences between two variables (Table 3). There were significant differences in the mean between methylparaben levels based on gender, age, BMI, and cosmetic use (*p* ≤ 0.001, *p* = 0.015, *p* = 0.011, and *p* ≤ 0.001, respectively). The methylparaben levels were higher in participants ≥ 33 years old compared to those < 33 years old. The number of overweight females positive for methylparaben was higher compared to that of normal-weight females and males, with the source of methylparaben originating from cosmetic use. There were no significant differences among other parabens.

### 3.1. EDCs with Age

Anatomical changes are known to occur in the kidneys of older individuals, such as fatty acid degeneration, irregular thickening of the basal membrane, increasing zones of tubular atrophy, and fibrosis. This causes a decrease in the glomerular filtration rate (GFR) and the effective renal plasma flow (ERPF). The low excretion rate in older people causes an accumulation of toxic substances inside the body [22].

### 3.2. EDCs with Gender

Humans are exposed to bisphenols primarily via oral, environmental, parenteral, and dermal routes [16]. Canned food, coffee pots, soda cans, and other kitchen plastics are believed to be major sources of oral bisphenol exposure [23]. In this study, men had higher BPS levels, because they used more plastic in their daily lives. Unlike bisphenol, the source of parabens is cosmetic and other personal care products; thus, higher levels of methylparaben were detected in women compared to men [20].

### 3.3. EDCs with BMI

In the metabolic pathway, bisphenols can interfere with several factors, of which one induces fibroblast differentiation into adipocytes, inhibits adiponectin, and affects glucose metabolism. In addition, parabens can promote adipogenesis and the adipogenic potential of parabens increases as the length of the linear alkyl chain increases [24]. Some studies mention an association between bisphenol levels and obesity in adults and children in the USA [25]. One study showed that the higher the bisphenol level, the higher the excretion rates among men, younger respondents, and those with an increasing waist circumference (*p* = 0.013) and weight (*p* = 0.003) [26]. Another study on parabens showed higher methylparaben and propylparaben levels in women with a BMI between 25 and 34.9 compared to those with a BMI between 18.5 and 24.9. These results are consistent with the results of this study [27].

### 3.4. EDCs with Cosmetic Use

Paraben esters and their salts are widely used as preservatives in cosmetics, toiletries, food, and pharmaceuticals. All women in this research study use cosmetics in their daily lives, which are the main source of parabens in this research. Parabens can enter the body through inhalation, ingestion, or via the skin. However, in this study, skin exposure was thought to be the largest route of entry [24]. After entering the body, the process of bioaccumulation or excretion to remove toxins outside the body will commence [20].

### 3.5. Limitations

This study only involved 150 samples from one geographic area, and it is necessary to study a wider population. It is also necessary to detect the levels of bisphenols and parabens in food, water, and personal products to make a health risk assessment.

## 4. Conclusions

This study successfully analyzed a valid, rapid, and simple extraction procedure of BPA analogues and parabens and subsequently detected them in human plasma by LC-MS/MS. Plasma from 150 healthy Malaysian volunteers was used to evaluate the levels of BPA analogues and parabens. Determination of the concentrations of BPA analogues and parabens from the subjects was satisfactorily performed using this proposed method. Our findings determined that BPF and methylparaben have the highest concentration compared to the other chemicals analyzed. Therefore, human beings from Malaysia are exposed to different BPA analogues and parabens, from the daily use of products and from the environment. Further research is needed with a larger and varied cohort to explore the presence of BPA analogues and parabens in the general population, in order to create a general risk assessment database.

## Figures and Tables

**Table 1:** BPA analogue and paraben detection.

Variable	*N* (%)	Mean ± SD (ng/mL)	Variable	*N* (%)	Mean ± SD (ng/mL)
BPAF		11.24 ± 1.96	Propylparaben		1.55 ± 0.91
Positive	36 (24)		Positive	66 (44)	
Negative	114 (76)		Negative	84 (56)	
BPS		13.03 ± 7.2	Ethylparaben		0.4 ± 0.55
Positive	16 (10.7)		Positive	48 (32)	
Negative	134 (89.3)		Negative	102 (68)	
BPB		—	Butylparaben		0.47 ± 0.26
Positive	0 (0)		Positive	20 (13.3)	
Negative	150 (100)		Negative	130 (86.7)	
BPF		34.87 ± 8.93	Methylparaben		7.7 ± 3.91
Positive	149 (99.3)		Positive	25 (16.7)	
Negative	1 (0.7)		Negative	125 (83.3)	

**Table 2:** Differences in BPS levels based on demographics and daily habits.

Variable	*N*	Mean	*p* value	Variable	*N*	Mean	*p* value
Gender				Plastic use			
Male	43	2.23	0.003	Yes	131	1.04	0.096
Female	107	1.06		No	19	3.79	
Age				Source of food			
<33 yo	109	1.03	0.004	Home cooking	91	2.15	0.25
≥33 yo	41	2.36		Dining out	59	0.21	
BMI				Source of drinking water			
Normal weight	87	0.79	≤0.001	Tap water	111	1.57	0.109
Overweight	63	2.22		Mineral water	39	0.87	

yo: years old.

**Table 3:** Differences in methylparaben levels based on demographics and cosmetic use.

Variable	*N*	Mean	*p* value	Variable	*N*	Mean	*p* value
Gender				BMI			
Male	43	0.512	≤0.001	Normal weight	87	0.93	0.011
Female	107	1.595		Overweight	63	1.77	
Age				Cosmetic use			
<33 yo	109	1.045	0.015	Yes	43	0.512	≤0.001
≥33 yo	41	1.921		No	107	1.595	

yo: years old.

## Data Availability

The excel data used to support the findings of this study are included within the supplementary information file (available here). The supplementary information file contains the raw data before analysis process by SPSS.

## Supplementary Materials

Supplementary Materials Supplementary information file contains raw data before the analysis process by SPSS.
